# Mechanochemical Engineered Sulfur Copolymers Onto Group 13 and Related Fillers Through Lewis Acid‐Base Pairing

**DOI:** 10.1002/advs.78222

**Published:** 2026-10-11

**Authors:** Chen Wang, Xiaoyang Shao, Zixuan Lei, Ruoqing Zhao, Xiaozhong Bao, Shuiping Zhou, Wen Ao, Zhenhua Wang

**Affiliations:** ^1^ State Key Laboratory of Flexible Electronics (LOFE) & Institute of Flexible Electronics (IFE) Northwestern Polytechnical University Xi'an China; ^2^ National Key Laboratory of Solid Rocket Propulsion Northwestern Polytechnical University Xi'an China; ^3^ Hubei Institute of Aerospace Chemical Technology Xiangyang Hubei China; ^4^ Key Laboratory of Science and Technology on High‐tech Polymer Materials Institute of Chemistry Chinese Academy of Sciences Beijing China

**Keywords:** group 13 and related fillers, mechanochemistry, radical copolymerization, sulfur copolymers, surface engineering

## Abstract

Group 13 and related fillers are cornerstone components for advanced composites, yet their severe interfacial incompatibility with organic matrices inherently limits their practical efficacy. Conventional surface modification strategies remain fundamentally constrained by a heavy reliance on hazardous solvents, matrix curing inhibition, and structural degradation of the fillers. Herein, we report a solvent‐free mechanochemical copolymerization strategy that establishes an universal, non‐destructive interfacial engineering paradigm for diverse Group 13 and related fillers (nano‐Al, B_4_C, and ZrB_2_). By directly architecting sulfur‐containing polymer coatings on filler surfaces, this approach elegantly exploits intrinsic Lewis acid–base pairing between electron‐deficient metal/metalloid sites and electron‐rich sulfur moieties. Mechanistically, the resulting electronic configurations and coordination chemistry are systematically elucidated via a synergistic combination of XPS, solid‐state ^11^B NMR, and DFT calculations, confirming the formation of robust S–Al and S–B coordinative networks. The extraordinary universality of this matrix‐agnostic platform is validated by incredible performance increments across three distinct frontiers, driving a staggering 666% surge in combustion enthalpy for nano‐Al fuels, a 33% ablation reduction for ZrB_2_ thermal shields, and concurrent ∼270% elevations in both strength and toughness for B_4_C structural composites. Ultimately, this environmentally benign protocol paves a versatile avenue toward the green synthesis of high‐performance hybrids.

## Graphical abstract

Green mechanochemical copolymerization was developed for universal interfacial modification of Group 13 and related fillers. By leveraging Lewis acid–base coordination to anchor sulfur‐containing polymers—validated via multiscale characterization and DFT—this versatile method boosts performance across liquid fuels, thermal‐shielding materials, and structural composites, establishing a sustainable paradigm for advanced hybrid material fabrication.

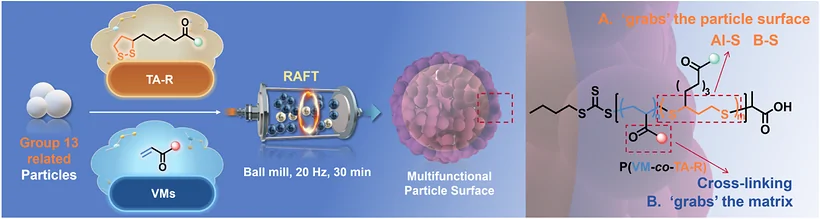

## 1 Introduction

Main Group 13 and related fillers, prominently exemplified by nano‐aluminum powder (nano‐Al, featuring a native surface Al_2_O_3_ layer), boron carbide (B_4_C), and zirconium diboride (ZrB_2_), have emerged as an exceptionally vital class of inorganic phases within the broad landscape of modern materials science and advanced engineering [1, 2, 3, 4]. Benefiting from the unique electronic configurations and intrinsic periodic attributes of Group 13 elements, these materials possess a remarkable combination of high thermal conductivity, exceptional mechanical hardness, robust neutron‐capturing cross‐sections, and extreme refractory stability [5, 6]. Consequently, their utility as high‐performance fillers transcend the limitations of any single host matrix, serving as critical enablers across a diverse spectrum of cutting‐edge technological fronts. Specifically, nano‐Al powders, leveraging its high energy density and the thermal dissipation capabilities of its core‐shell structure, is widely utilized in advanced energetic composites [7, 8]. B_4_C, distinguished by its immense neutron absorption capability and outstanding hardness, represents the premier candidate for nuclear radiation shielding and ballistic armor composites [9, 10]. Meanwhile, ZrB_2_ serves as an indispensable material for extreme ablation environments such as the leading edges of hypersonic vehicles and rocket propulsion nozzles [11, 12]. Integrating these versatile fillers into multi‐component matrices provides a powerful paradigm for fabricating advanced hybrid materials with customized thermomechanical profiles [13, 14, 15].

Despite the profound potential of these Group 13 and related fillers, their practical efficacy within liquid or polymer matrices is severely hindered by poor interfacial compatibility. Owing to the high surface energy and intrinsically hydrophilic nature of the inorganic surfaces (such as the native oxide shell on nano‐Al), they exhibit a strong propensity to severely agglomerate within hydrophobic matrices, inducing localized stress concentrations [16, 17]. Consequently, surface modification of these fillers is of paramount importance to tailor their surface chemistry, optimize phase dispersion, and establish robust interfacial bonding. However, achieving efficient and universal surface modification remains a challenge, as traditional methods present distinct bottlenecks. Physical blending or surfactant coating suffers from weak interfacial adhesion and is highly prone to phase separation under thermal or mechanical stress [18, 19, 20, 21, 22]. Conversely, traditional chemical grafting via silane coupling agents or aggressive acid etching, while offering stronger chemical bonding, frequently relies on hazardous organic solvents, exhibits poor chemical universality across structurally diverse Group 13 and related compounds, and risks compromising the intrinsic crystal lattice or reactive core of the underlying fillers [23, 24, 25, 26, 27]. While biomimetic strategies like polydopamine coating offer interfacial adhesion, their practical implementation in advanced composite manufacturing remains severely constrained by the curing kinetics of the host resin matrices, leading to incomplete or uncontrolled matrix crosslinking [28, 29, 30, 31, 32].

To overcome these critical bottlenecks, herein, we report a versatile strategy to engineer the grain boundaries and surfaces of Group 13 and related fillers via solid‐state mechanochemical free‐radical polymerization [33, 34, 35, 36, 37, 38, 39, 40, 41, 42, 43, 44], achieving the in situ construction of protective and functional sulfur‐containing polymers directly on the filler surfaces (Scheme 1) [45, 46, 47, 48]. This solid‐state mechanochemical approach offers paradigm‐shifting advantages over conventional liquid‐phase protocols. First, by entirely eliminating the reliance on solvent media, it completely bypasses the monomer incompatibility typically induced by solvation effects or solubility mismatches in solution. Second, it introduces a highly adaptable and modular platform [49, 50, 51], allowing for the precise selection and tailor‐made matching of functional co‐monomers to topologically and chemically align with the specific target curing resin matrix system [52, 53, 54, 55]. Mechanistically, this interfacial design paradigm seamlessly exploits the intrinsic Lewis acid‐base interactions between the electron‐deficient Group 13 elements (acting as Lewis acids, such as the Al sites in the surface oxide layer) and the electron‐rich sulfur moieties of the mechanochemically grafted polymer coatings (acting as Lewis bases). This localized chemical affinity establishes an ultra‐robust, non‐destructive interfacial bonding layer without altering the pristine crystal structures or the core reactivity of the fillers. Crucially, the universal applicability of this interfacial design has been successfully validated across a diverse spectrum of curing resin matrices. The resulting composites exhibit comprehensive performance enhancement, characterized by significantly enhanced mechanical strength, superior flame retardancy, and outstanding ablation resistance under extreme thermal and mechanical environments [56]. Overall, this work offers a versatile, eco‐friendly, and highly tunable Methodology for the structural design and green manufacturing of advanced polymer‐inorganic hybrid materials [57].

**SCHEME 1:**
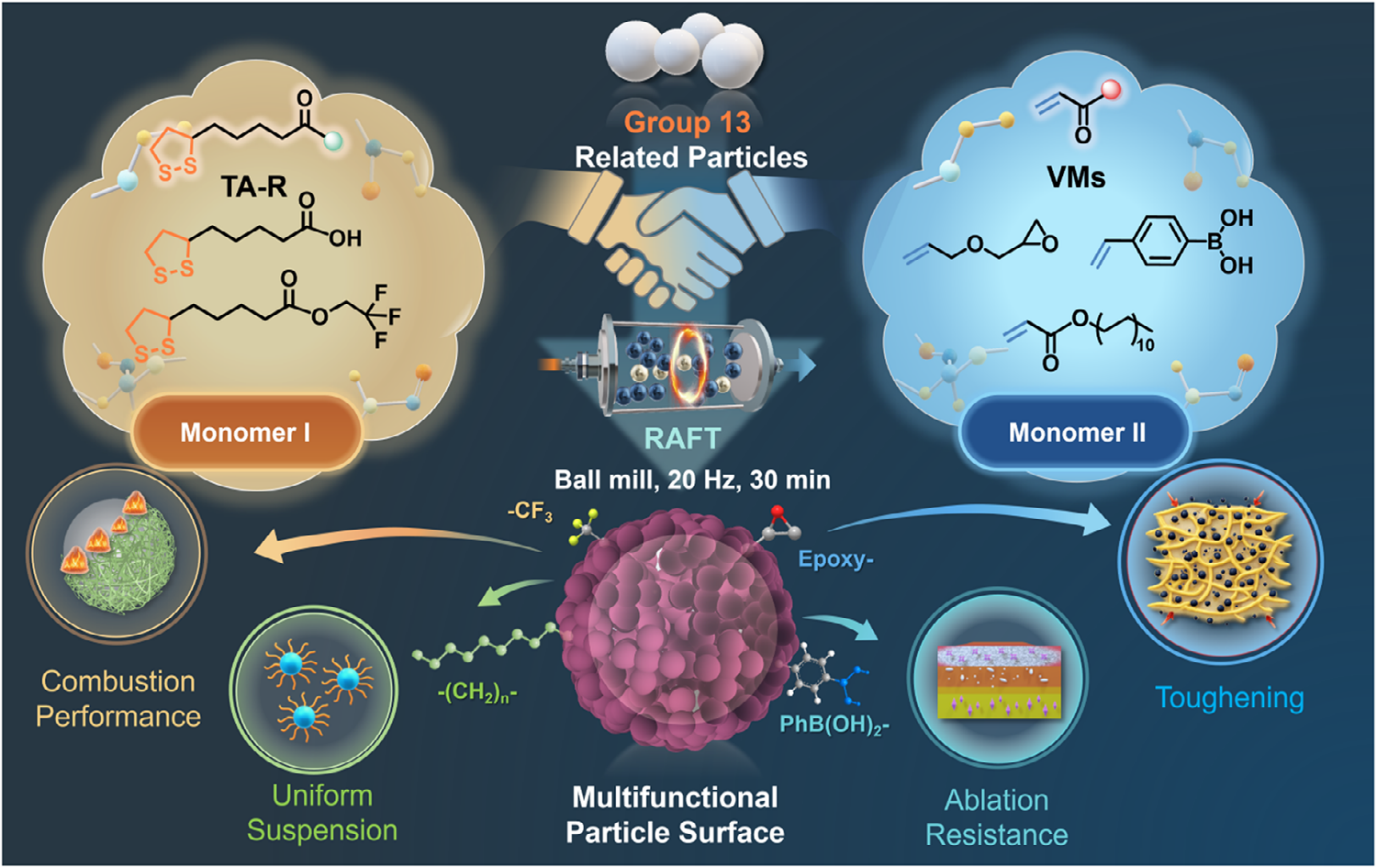
Mechanochemically triggered dual‐polymer coatings for universal particle surface engineering.

## 2 Results and Discussion

### 2.1 Aerobic Mechano‐RAFT Copolymerization by Ball‐Milling

For the customized surface engineering of Group 13 and related fillers, a solvent‐free mechano‐oxygen synergistic copolymerization strategy was empolyed. This methodology uniquely exploits lipoic acid (TA) and vinyl monomers as the hub monomers, mediated by amine‐borane complexes under ball milling without degassing. The radical‐induced ring‐opening in TA drives provides electron‐rich sulfur moieties for Lewis acid‐base recognition, the vinyl monomers allow for the modular customization to endow its compatibility with diverse matrices. Taken advantage of mechano‐oxygen synergy, this system efficiently constructs customized polymeric coatings directly on filler surfaces. To verify its feasibility, aerobic mechano‐RAFT copolymerization of TA and its TA derivatives (2,2,2‐trifluoroethyl lipoate, TATF; phenethyl lipoate, TABN; etc.) with vinyl monomers (*n*‐butyl acrylate, *n*BA; or allyl glycidyl ether, AGE) was conducted using BTPA as the chain transfer agent and Et_3_B‐PyOMe as the mechanolabile initiator (Figure 1a). Ball milling (1.5 h, 30 Hz) successfully transformed the transparent liquid mixtures into highly viscous fluids. ^1^H NMR and GPC profiles confirmed efficient copolymerization: the TA/*n*BA system yielded conversions of 38.1% (TA) and 42.1% (*n*BA) with an *M*_n_ of 9,100 g/mol and a dispersity (*Đ*) of 1.99 (Figure 1b, Table S1, Entry 1), while the TA/AGE system reached 23.3% (TA) and 24.3% (AGE) (*M*_n_ = 15,200 g/mol, *Đ* = 1.85; Table S1, Entry 2). The general applicability was further validated via the successful copolymerization of various TA derivatives bearing distinct side groups with *n*BA (Figure 1b, Figure S1a, Table S1, Entries 3–5). This method is equally applicable to the copolymerization of solid vinyl monomers (NIPAM) with lipoic acid derivatives (Table S1, Entries 9–13). The TABn/NIPAM system achieved conversions of 25.3% (NIPAM) and 33.3% (TABn), with an *M*_n_ of 3,900 g/mol and a dispersity (*Đ*) of 1.91 (Figure 1b, Table S1, Entry 10). Conversely, non‐milled control experiments yielded negligible conversions (Figure S1b, Table S1, Entries 6–8), confirming that the copolymerization is driven by mechanical activation (Figure S2–10).

**FIGURE 1:**
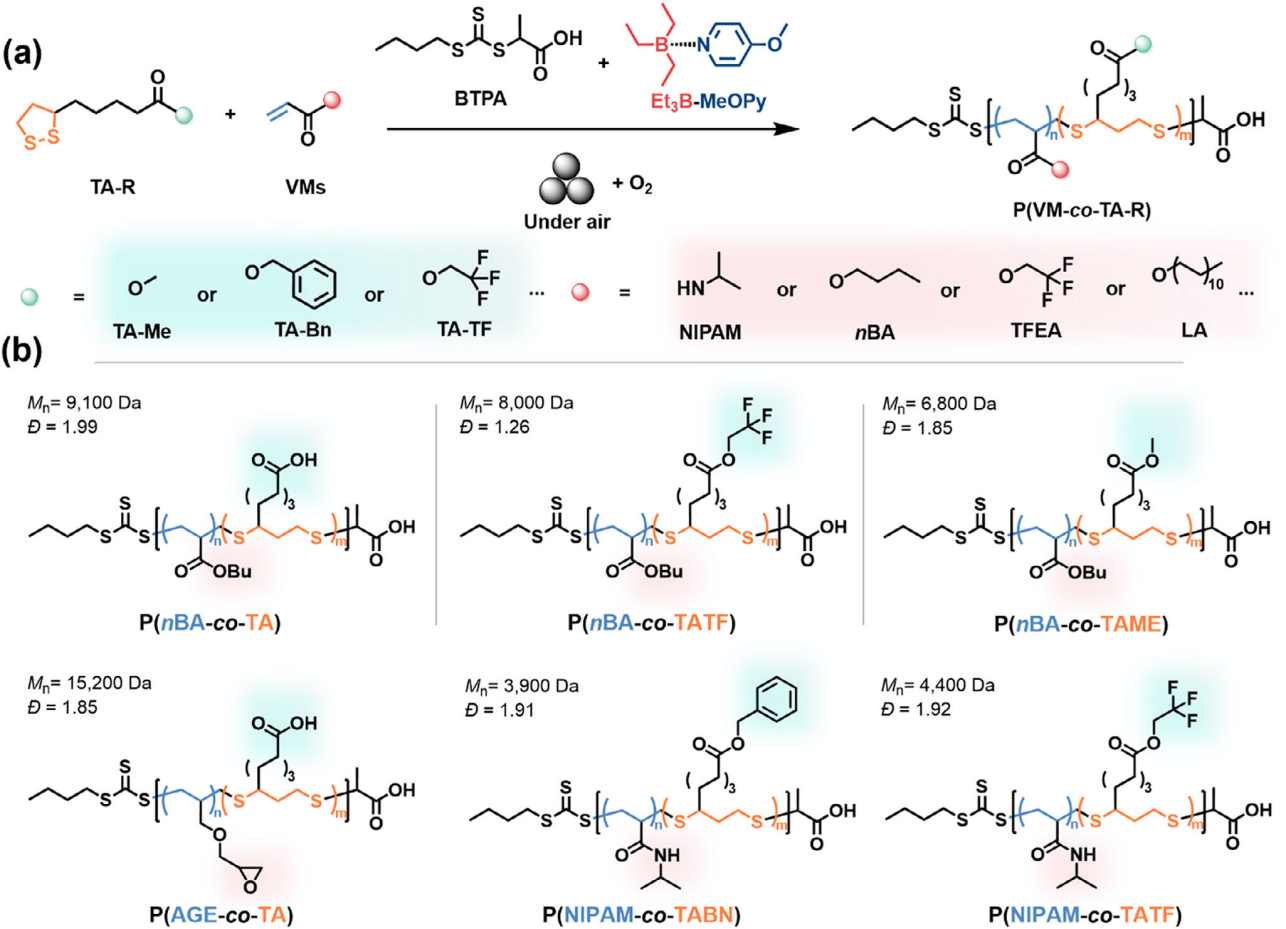
Aerobic mechano‐RAFT driven by ball‐milling. (a) Schematic copolymerization of TA‐R and VMs. (b) Representative structures and polymerization data of copolymers.

### 2.2 Preparation and Characterization of nAl and m‐nAl

Building upon the successful feasibility validation of mechanochemical copolymerization, nano‐aluminum powder (nAl) was initially selected as a model inorganic filler to evaluate the efficacy of this solid‐state mechanochemical modification strategy. Characterized by an exceptionally high specific surface area, shortened mass diffusion distances, and elevated chemical reactivity, nAl represents a highly promising next‐generation energetic fuel within the landscape of nanofluid fuels. Dispersing nAl into hydrocarbon liquid matrices (e.g., RP‐3) substantially enhances volumetric energy density, accelerates flame propagation, and improves overall combustion efficiency, thereby opening new frontiers for high‐performance propulsion systems. Nevertheless, the practical exploitation of pristine nAl is constrained by its severe agglomeration, poor colloidal stability in fluid media, and uncontrollable interfacial reactions. To address these long‐standing challenges, fluorinated thiotic acid monomers were mechanochemically copolymerized with long‐alkyl‐chain vinyl monomers to construct a protective and matrix‐compatible polymer shell (PLA‐*co*‐PTATF) directly on the nAl surfaces, yielding the modified energetic filler denoted as m‐nAl (Figure 2a).

**FIGURE 2:**
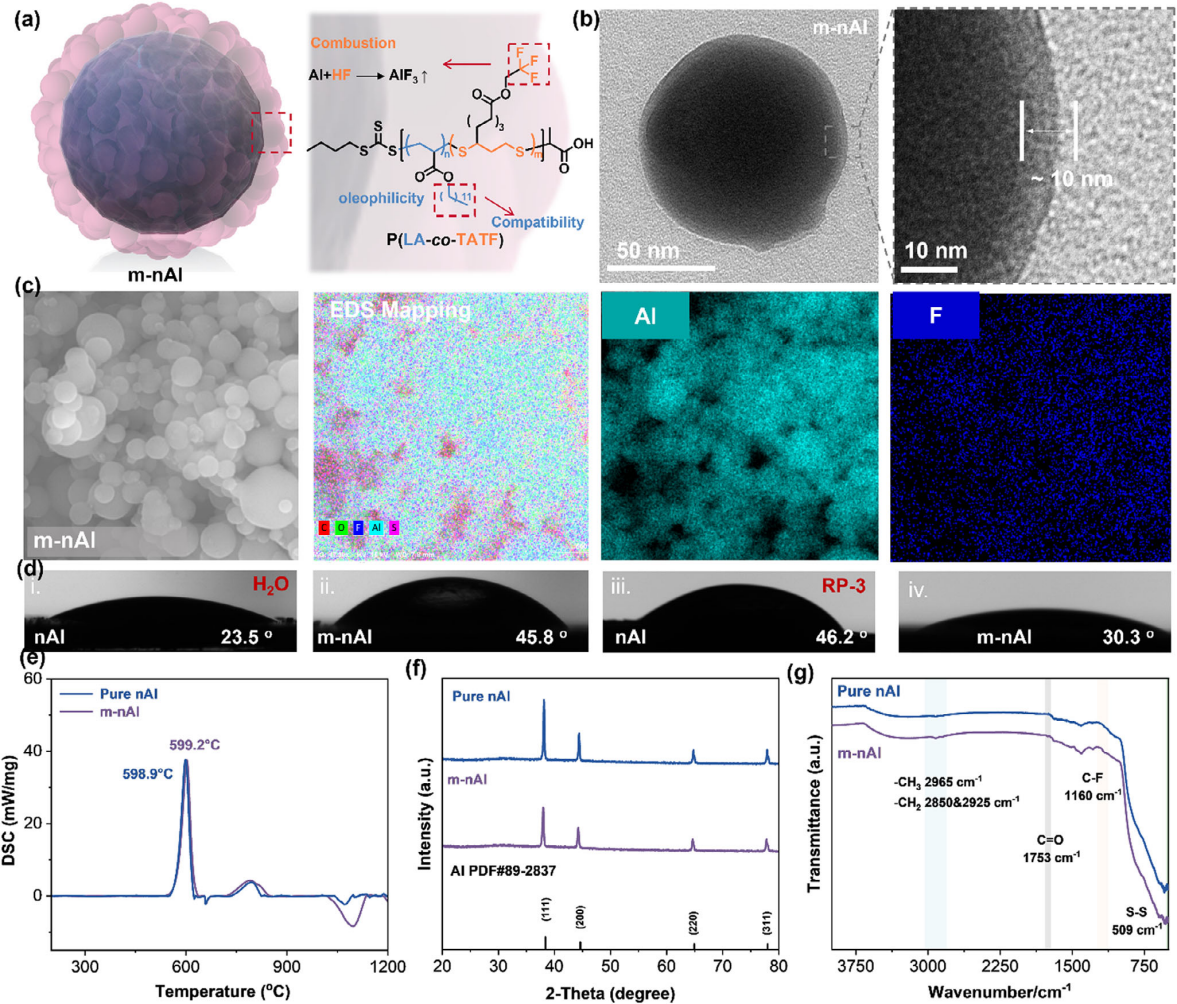
Microstructure, surface properties, and thermal performance characterization of pristine and modified nano‐aluminum powders. (a) Schematic illustration of P(LA‐*co*‐TATF) modified nano‐aluminum powder, (b) TEM images, (c) SEM‐EDS mappings, (d) static contact angles to water and RP‐3, (e) DSC curves, (f) XRD patterns and (g) FT‐IR spectras of pristine nAl and m‐nAl.

As illustrated in Figure 2, the structural morphology and physicochemical attributes of pristine nano‐aluminum (Pure nAl) and the copolymer‐functionalized counterpart (denoted as m‐nAl) were systematically evaluated. Transmission electron microscopy (TEM) imaging of m‐nAl (Figure 2b) reveals that the nanoparticles perfectly retain their well‐defined, intact spherical morphology while being seamlessly encapsulated by a continuous, highly conformal polymer shell (∼10 nm in thickness) that serves as a robust physical barrier to mitigate uncontrolled pre‐oxidation and suppress the particle agglomeration. This uniform coverage is corroborated by scanning electron microscopy coupled with energy‐dispersive X‐ray spectroscopy (SEM‐EDS) elemental mapping (Figure 2c, Figure S11), which demonstrates excellent macro‐scale dispersibility and a highly homogeneous spatial distribution of C, O, F, and Al signals across the inorganic surfaces. Surface energetic evolution quantified via static contact angle measurements (Figure 2d) demonstrates a reversed wettability profile—where the water contact angle increases from 23.5° to 45.8° and the RP‐3 aviation kerosene contact angle drops sharply from 46.2° to 30.3°—which is exceptionally advantageous for ensuring long‐term colloidal stability within hydrocarbon liquid nanofluid fuels. Furthermore, DSC measurements under an oxidative atmosphere (Figure 2e) show a preserved Al core melting endotherm at approximately 599°C, together with a pronounced high‐temperature oxidation exotherm, resulting in a 666% increase in combustion enthalpy relative to pristine Al powder. This enhancement is closely related to the interfacial structure of the coating: the ∼10 nm coating thickness observed by TEM, the uniform C/F/S distribution in the EDS maps, and the attenuation of the Al signal to 56.3% collectively indicate the formation of a continuous F‐rich interfacial layer on the particle surface. Upon heating, fluorine‐containing fragments released from the coating can react with the outer Al/Al_2_O_3_ layer to form volatile AlF_3_, thereby fluorinating and disrupting the refractory alumina shell. This interfacial fluorination weakens the passivation barrier, exposes more fresh and active Al, and facilitates oxygen access to the metal core, ultimately intensifying the high‐temperature oxidation exotherm. This non‐destructive modification paradigm is verified by X‐ray diffraction (XRD) patterns (Figure 2f), which display well‐resolved reflections at 2θ ≈ 38.4°, 44.7°, 65.1°, and 78.2° corresponding precisely to the standard face‐centered cubic lattice of Al (*PDF#89‐2837*) without introducing any crystalline impurity phases. Finally, Fourier‐transform infrared (FT‐IR) spectroscopy (Figure 2g) provides definitive molecular evidence of successful grafting, exhibiting distinct vibrational fingerprints at 2965 cm^−1^ (‒CH_3_ stretching), 2925/2850 cm^−1^ (‒CH_2_‒ stretching), 1753 cm^−1^ (C = O stretching), 1160 cm^−1^ (C‒F stretching), and 509 cm^−1^ (S‒S linkage stretching).

### 2.3 Nanofluid Fuel (nAl in RP‐3)

To quantify the benefit of the surface modification, the colloidal dispersion stability of the surface‐engineered m‐nAl was systematically evaluated against pristine nAl and oleic acid‐stabilized nAl (nAl+OA) in RP‐3 aviation kerosene (Figure 3a,b). Initially (0 h), all configurations formed completely homogeneous, opaque black suspensions. However, severe phase separation occurred in the pristine nAl system after 3.0 h of static settling, while the nAl+OA counterpart displayed only marginal stabilization [58]. In stark contrast, the m‐nAl nanofluid maintained flawless suspension uniformity. After an extended 13.0 h standing period, pristine nAl underwent near‐complete sedimentation, leaving a transparent supernatant, and the nAl+OA system exhibited profound precipitation. Remarkably, the m‐nAl sample retained a dark, highly homogeneous suspension with minimal sediment, visually demonstrating superior anti‐sedimentation capability driven by the enhanced steric stabilization and oleophilic compatibility imparted by the long‐alkyl‐chain copolymer shell. These visual observations were quantitatively validated by tracking sedimentation kinetics using real‐time fluorescence spectroscopy, capitalizing on the intrinsic emission profile of pure kerosene at 400 nm (Figure 3c). Effective shielding of this optical signal corresponds to well‐suspended nanoparticles, whereas particle depletion in the upper layer causes a concomitant rise in fluorescence intensity. After a 2 h aging period, the pristine nAl fluid exhibited the highest upper‐layer fluorescence intensity, confirming severe particle depletion (Figure 3d, Figure S12). The nAl+OA sample showed intermediate intensity, reflecting limited dispersion enhancement. Conversely, the m‐nAl system displayed the lowest fluorescence intensity, corroborating prolonged particle suspension and superior optical shielding. This quantitative profile was further corroborated by digital grayscale analysis of the sedimentation images (Figure S13), yielding a variation trend in perfect agreement with the optical fluorescence data and confirming the robust long‐term colloidal longevity achieved by the mechanochemical coating paradigm. Beyond achieving colloidal stability, the engineered interfacial shell delivered exceptional advancements in droplet combustion dynamics, captured via high‐speed imaging. The m‐nAl nanofluid achieved a condensed burnout timeline of 2.0 s compared to 2.7 s for pristine nAl, leaving a remarkably clean silicon carbide (SiC) support fiber with minimal oxide residue, signifying highly complete and accelerated combustion (Figure 3e). Temporal tracking of droplet size evolution revealed that the incorporation of pristine nAl induced violent, irregular dimensional oscillations due to localized particle agglomeration and uneven boiling (Figure S14a–c), a phenomenon successfully mitigated in the m‐nAl system, which displayed significantly suppressed fluctuations. Quantitative linear fitting determined that the addition of pristine nAl reduced the combustion rate constant from 0.66 mm^2^/s (pure kerosene) to 0.63 mm^2^/s due to agglomeration‐induced heat transfer degradation. Strikingly, the m‐nAl nanofluid achieved an elevated combustion rate constant of 0.82 mm^2^/s (Figure 3f). This substantial kinetic acceleration is directly ascribed to the pre‐engineered fluorine constituents within the polymer shell; during initial heating, the aggressive interaction between fluorine and the passivating aluminum oxide shell induces the sublimation of volatile m‐nAl, effectively stripping away the oxide layer to continuously expose the active aluminum core.

**FIGURE 3:**
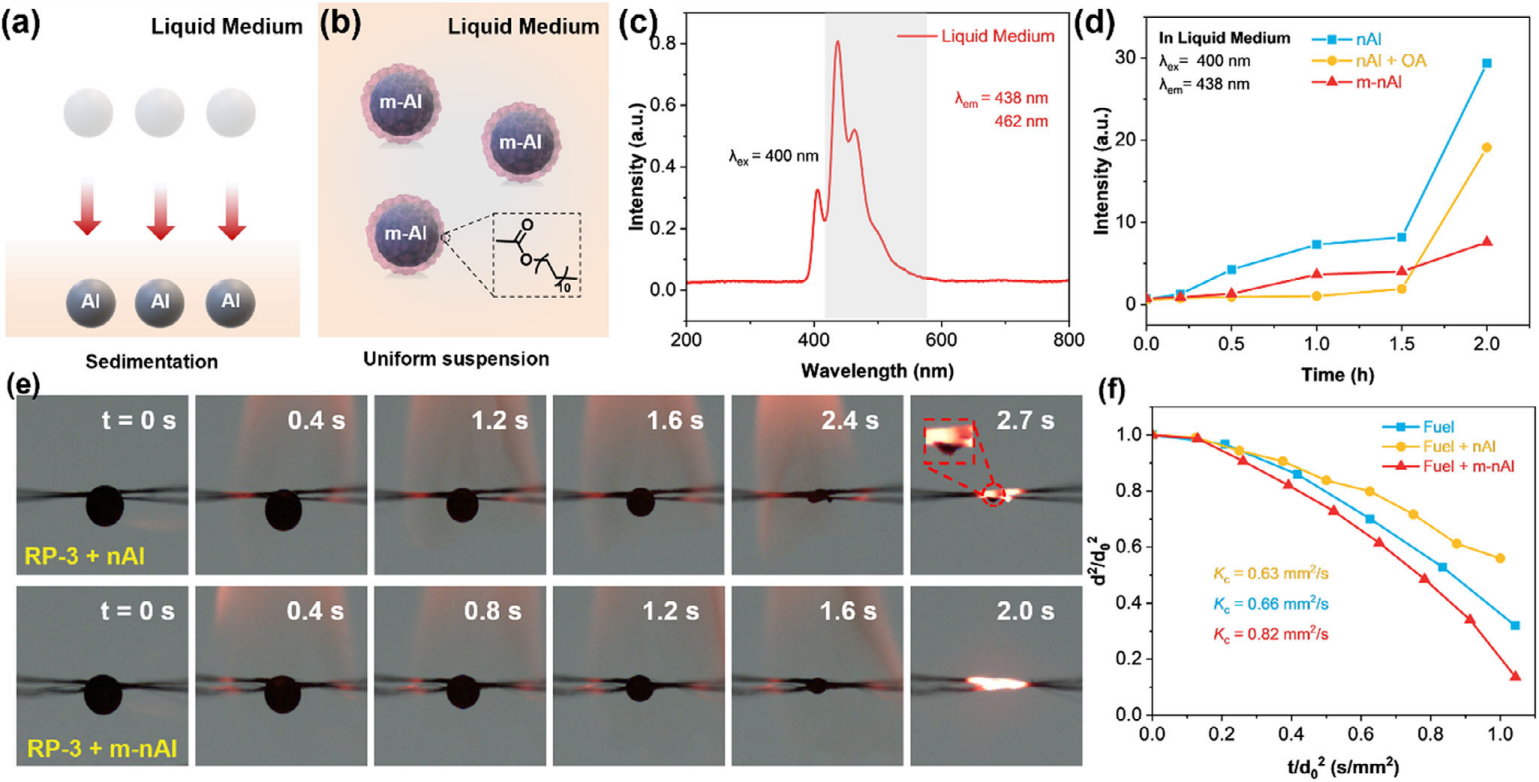
Characterization of combustion performance and suspend stability of nano‐sized aluminum powder within RP‐3. Dispersion behavior of (a) nAl and (b) m‐nAl in RP‐3. (c) Fluorescence emission spectrum of pure RP‐3 kerosene. (d) Fluorescence intensity of the supernatant of different nanofluid fuels as a function of settling time. (e) High‐speed camera images of droplet combustion process of RP‐3‐based nanofluid fuels, and (f) droplet combustion kinetics curves of different RP‐3‐based nanofluid fuels.

### 2.4 Preparation and Characterization of ZrB_2_, B_4_C, m‐ZrB_2_ and m‐B_4_C

To demonstrate the universality of this paradigm, the solid‐state strategy was extended from nano‐aluminum to boron‐containing ceramics (ZrB_2_ and B_4_C), leveraging the universal affinity of sulfur for electron‐deficient Al and B sites [59]. For the ultra‐high‐temperature ceramic ZrB_2_, copolymerization of thiotic acid (TA) with vinylphenylboronic acid (VPBA) successfully generated ZrB_2_@PVPBA‐*co*‐PTA (m‐ZrB_2_) (Figure 4a), establishing a porous encapsulation layer that bridged loose particles into secondary agglomerates. Similarly, for B_4_C, mechanochemical copolymerization of TA with allyl glycidyl ether (AGE) yielded B_4_C@PAGE‐*co*‐PTA (m‐B_4_C) (Figure 5a); scanning electron microscopy (SEM) imaging (Figure S16) revealed that the sharp polyhedral edges of the pristine crystals were passivated by a tightly bound, wrinkled polymer coating designed to improve epoxy matrix compatibility. X‐ray diffraction (XRD) patterns (Figures S17) confirmed the non‐destructive nature of this mechanochemical process, as the structural integrity and characteristic reflections of both B_4_C (PDF #01‐086‐1118, with a trace manganese impurity at 2θ ≈ 29.5°) and ZrB_2_ (PDF #00‐034‐0423) remained perfectly preserved. Finally, while the pristine ceramics exhibited featureless, thermally stable differential scanning calorimetry (DSC) baselines up to 500 °C, the functionalized architectures displayed distinct polymeric decomposition endotherms at 249.8 °C (m‐B_4_C) and 256.9 °C (m‐ZrB_2_) (Figures S18), providing unequivocal proof of successful universal interfacial encapsulation. The FT‐IR spectra also confirmed that all three powders were coated with the sulfur‐containing copolymer (Figure S19). XPS survey spectra were employed to characterize the surface coating of B_4_C and ZrB_2_ powders. The results showed that after coating modification, the sulfur content on the B_4_C and ZrB_2_ surfaces increased from 0 to 3.8% and 3.1%, respectively, confirming the successful encapsulation by the sulfur‐containing copolymer (Figure S20, Table S2).

**FIGURE 4:**
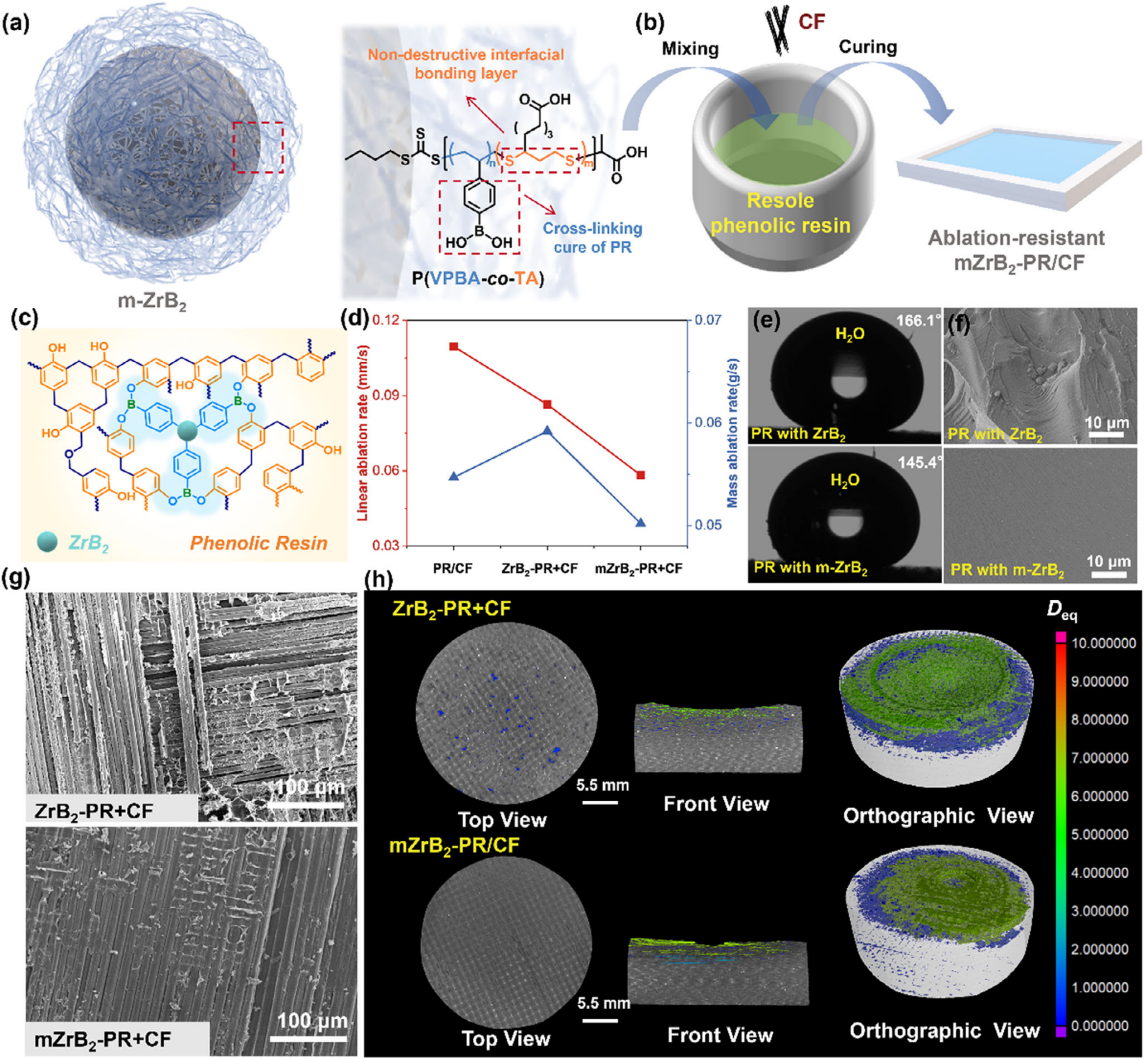
(a‐b) Schematic illustration of P(VPBA‐*co*‐TA) modified ZrB_2_. (c) Schematic illustration of interfacial interaction between modified ZrB_2_ and phenolic resin matrix. (d) Linear ablation rate and mass ablation rate of different PR/CF composites. (e) Water contact angle measurements of ZrB_2_/PR and modified ZrB_2_/PR composites. (f) Fracture surface SEM images of ZrB_2_/PR and modified ZrB_2_/PR composites. (g) Surface morphology SEM images of Z‐PR/CF and mZ‐PR/CF composites after ablation and (h) CT images from different perspectives.

**FIGURE 5:**
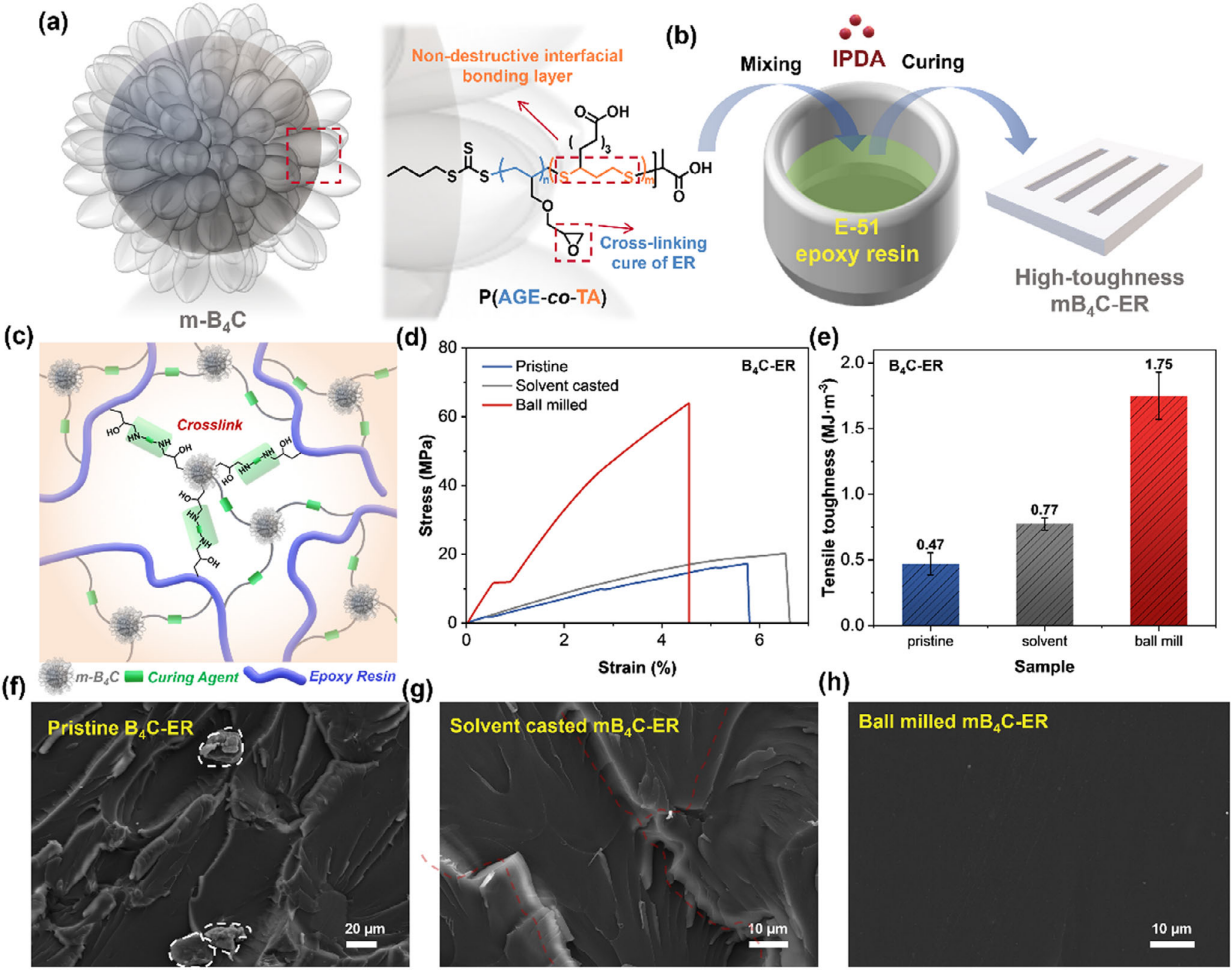
(a‐b) Schematic illustration of P(AGE‐*co*‐TA) modified B4C. (c) Schematic illustration of the toughening mechanism of modified B_4_C in epoxy resin. (d‐e) Stress‐strain and tensile toughness of ER composites filled with unmodified and modified B_4_C. (f‐h) SEM images of fracture surfaces of B_4_C/ER.

To address the inherently weak interfacial compatibility between the ultra‐high‐temperature ceramic filler and the phenolic resin matrix, ZrB_2_ particles were encapsulated with a VPBA and TA copolymer shell (Figure 4b). As schematically illustrated in Figure 4c, the pendant phenylboronic acid functionalities within the pre‐engineered coating layer covalently participate in the cross‐linking cure of the phenolic resin (PR), converting the traditionally weak ZrB_2_/PR physical interface into a chemically interactive interphase. This interfacial tailoring directly modulates the surface energy and dispersion state of the ceramic phase. Static water contact angle measurements (Figure 4e) show a decrease from 166.1° for pristine ZrB_2_/PR to 145.5° for ZrB_2_/PR, providing clear evidence of enhanced resin wettability and matrix infiltration. This optimization is microscopically validated by fracture surface SEM imaging (Figure 4f); while the unmodified composite suffers from severe particle agglomeration and extensive interfacial debonding, the modified counterpart exhibits a more uniform and compact microstructure, characterized by better‐dispersed ceramic particles and fewer visible interfacial gaps within the PR matrix. The improved interfacial homogeneity contributes to the enhanced ablation resistance of the carbon fiber‐reinforced composites under oxyacetylene flame exposure. Oxyacetylene ablation testing (Figure 4d) demonstrates that the modified carbon fiber‐reinforced composite (mZ‐PR/CF) achieves a linear ablation rate of just 0.06 mm/s and a mass ablation rate of 0.05 g/s, representing a remarkable 33% reduction in linear erosion compared to the unmodified baseline (Z‐PR/CF: 0.09 mm/s and 0.06 g/s). Post‐ablation SEM images further show severe matrix recession and extensive carbon‐fiber exposure in Z‐PR/CF, whereas mZ‐PR/CF retains a more continuous ceramic‐containing surface layer (Figure 4g). X‐ray computed tomography (CT) scanning further reveals the effect of interfacial modification on the internal damage evolution after ablation (Figure 4h). Z‐PR/CF shows deep and irregular internal cavities. In contrast, mZ‐PR/CF preserves a more continuous internal framework with reduced damage penetration. This difference suggests that the improved ZrB_2_/PR interfacial compatibility helps maintain the integrity of the ceramic–char framework, thereby limiting the inward extension of ablation‐induced structural damage.

To evaluate the practical reinforcing and toughening efficacy of the functionalized ceramic architectures, the as‐prepared m‐B_4_C particles were incorporated into an epoxy resin (ER) matrix (Figure 5b). The proposed solvent‐free mechanochemical (ball‐milled) strategy was systematically benchmarked against both pristine B_4_C and a conventional liquid‐phase solvent‐cast route (Figure 5c). The pre‐engineered epoxy functionalities residing within the copolymer shell are specifically designed to covalently co‐cure with the ER matrix, transforming a traditionally weak physical interface into a robust, chemically bonded network. Tensile evaluation reveals a dramatic divergence in mechanical performance (Figure 5d). The pristine B_4_C/ER composite exhibits a meager tensile strength of ∼17 MPa, and the counterpart based on solution modification yields only a marginal increment to ∼20 MPa. In sharp contrast, the ball‐milled modified composite delivers a remarkable tensile strength of ∼65 MPa, accompanied by a vastly enhanced elastic modulus. This simultaneous increment in strength and extensibility translates to a profound boost in tensile toughness, calculated from the integrated area beneath the stress‐strain curves (Figure 5e). The mechanochemically optimized composite achieves a toughness of 1.75 MJ·m^−3^, representing a staggering 272% improvement over the pristine control (0.47 MJ·m^−3^), whereas the solvent‐cast method only yields a moderate value of 0.77 MJ·m^−3^. These mechanical performance leaps are strongly corroborated by cross‐sectional fractography via SEM observations (Figure 5f–h). The fracture surface of the pristine composite displays severe B_4_C agglomeration and extensive interfacial debonding, indicating poor stress transfer and premature structural failure under external loading. While the counterpart sample based on solution modification manifests localized crack deflection, it fails to eliminate interfacial gaps and clustering defects. Conversely, the ball‐milled modified composite presents a highly continuous, tortuous, and dense fracture morphology completely devoid of visible particle aggregates or void spaces. This microscopic homogeneity confirms that the solvent‐free mechanochemical strategy successfully eliminates particle clustering and establishes impeccable filler‐matrix adhesion. Consequently, external stress is efficiently transferred across the interface, allowing the well‐dispersed ceramic phase to fully activate energy‐dissipation mechanisms—such as crack pinning, deflection, and bridging—and successfully unlock simultaneous enhancements in both composite strength and toughness.

### 2.5 Interfacial Adsorption Mechanism of Sulfur‐Containing Copolymer on nAl, ZrB_2_ and B_4_C Surfaces

To gain fundamental quantum‐chemical insights into the interfacial reinforcement mechanism, density functional theory (DFT) calculations were systematically performed using Materials Studio 2020 (detailed computational configurations are provided in the Supporting Information). The interfacial binding energy (*E*_bind_) was utilized to quantify the adhesion strength for the organic–inorganic heterostructure boundary. To simplify the calculation while capturing the core coordination chemistry, poly(methyl acrylate) (PMA, *DP* = 2) and polythioctic acid (PTA, *DP* = 2)were employed as representative oligomeric models. This structural pairing was strategically devised to decouple and isolate the electronic impacts of a conventional all‐carbon framework (PMA) versus a sulfur‐rich backbone (PTA) on their respective interfacial chemisorption and coordination behaviors. The binding energy was calculated using the following equation:

(1) $$E_{\mathrm{bind}}=-\left(E_{\mathrm{total}}-E_{1}-E_{2}\right)$$

where *E*_bind_ is the binding energy between two interfaces, *E*_total_ is the total energy of the mixed system, *E*_1_ is the energy of the lower interface in the mixed system, and *E*_2_ is the energy of the upper interface in the mixed system; all energies are expressed in eV.

Molecular simulation results clearly compare the interfacial interactions between the two polymer layers and the surfaces of nAl/ZrB_2_ substrates. The left side illustrates the systems of all‐carbon backbone poly(methyl acrylate) (PMA, DP = 2) interacting with Al_2_O_3_ and ZrB_2_ substrates, whose calculated binding energies are 0.09 and 0.74 eV, respectively. The right side shows the simulation of sulfur‐containing polythioctic acid (PTA, DP = 2) adsorbed on Al_2_O_3_ and ZrB_2_ surfaces, with the binding energies increased to 0.48 and 1.36 eV compared with the all‐carbon backbone polymer PMA. These results demonstrate that, relative to the all‐carbon backbone of PMA, the sulfur‐containing backbone of PTA introduces much stronger chemical interactions with nAl and ZrB_2_ surfaces via S‐Al coordination and S‐B coordination (Figure 6a, Table S3).

**FIGURE 6:**
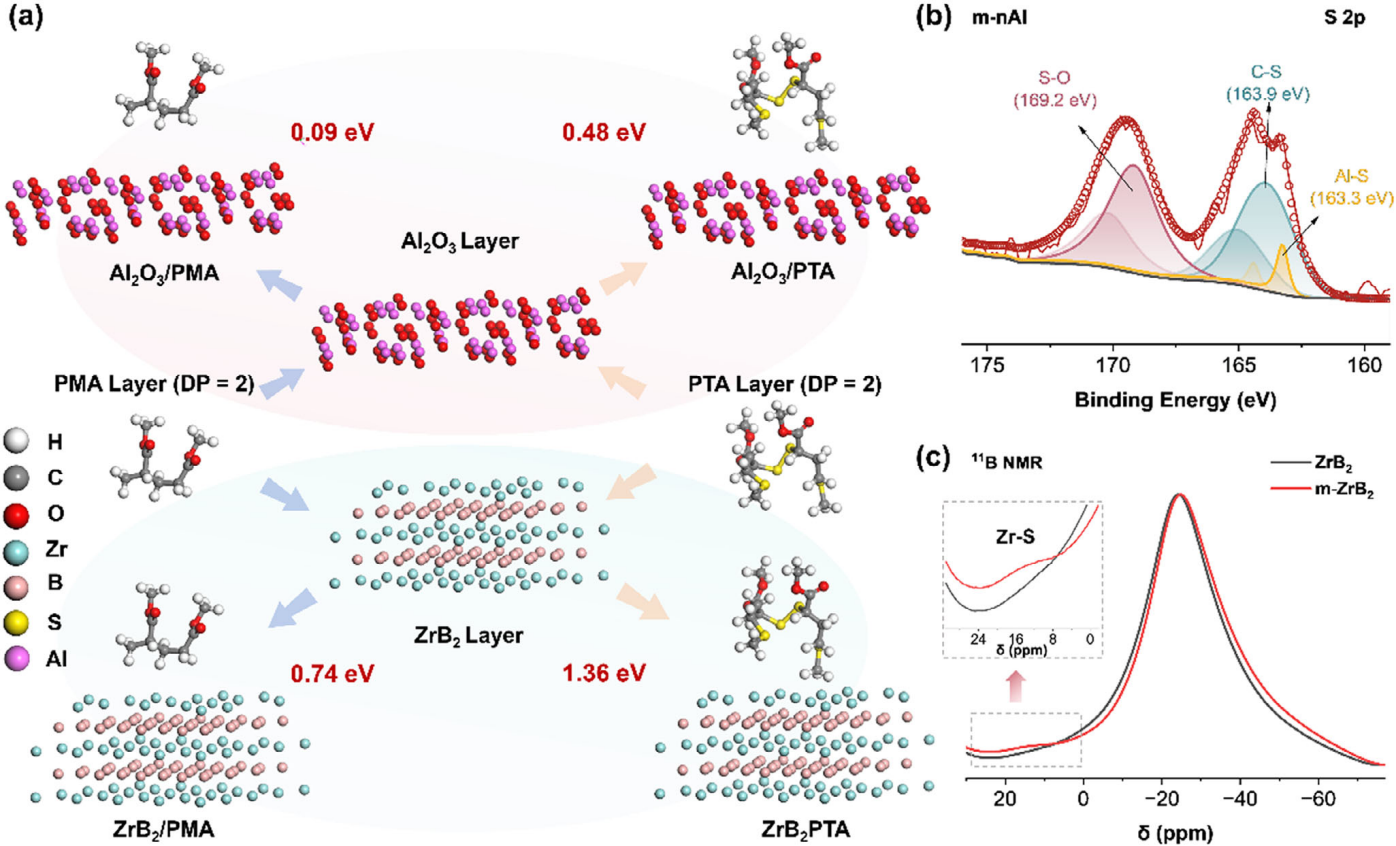
(a) The construction process and binding energy of ZrB_2_ with PMA or PTA interface layer, (b) typical high‐resolution XPS spectra of S 2p for m‐nAl and (c) ^11^B NMR spectra of ZrB_2_ and m‐ZrB_2_.

To further reveal the interaction mechanism between sulfur‐containing polymer coating and nano‐aluminum powder, X‐ray photoelectron spectroscopy (XPS) characterizations were carried out, and Lorentzian functions were used to fit the spectral data. Figure S21 displays the XPS survey spectra of pristine nano‐Al and m‐nAl. For the m‐nAl sample, characteristic peaks located at 161, 225, 685 and 832 eV are assigned to S 2p, S 2s, F 1s and F Auger peak, respectively. The surface coverage of sulfur‐fluorinated copolymer on aluminum powder can be roughly calculated according to the variation of surface atomic percentage of Al obtained from XPS survey spectra. The results show that the surface atomic percentage of Al in uncoated nano‐aluminum powder is 19.0%, which decreases to 8.3% after coating with P(LA‐*co*‐TFTA), corresponding to a surface coverage of 56.3%. It is worth noting that the 56.3% value represents a semi‐quantitative XPS‐derived estimate of the degree of coating, rather than an absolute measure of geometric surface coverage. Figure S22 present the high‐resolution spectra of Al 2p, F 1s, S 2p and C 1s for pristine nAl and m‐nAl, respectively. For the Al 2p spectrum of pristine nano‐Al, the peaks at 72.0 and 74.5 eV correspond to elemental aluminum (metallic Al) and Al‐O bond of alumina, respectively. By contrast, new characteristic peaks of Al‐F bond at 76.1 eV and Al‐S bond at 74.9 eV emerge in the Al 2p spectrum of m‐nAl. In the S 2p spectrum of m‐nAl, the spin‐orbit splitting peaks near 163.9 eV are attributed to C‐S bonds, the peak at 169.2 eV corresponds to S‐O bonds, and an additional peak at 163.3 eV verifies the formation of Al‐S bonds again (Figure 6b). The above results confirm that the sulfur‐containing polymer coating can tightly bind to the aluminum matrix and its surface alumina layer via Al‐S and Al‐F interfacial interactions.

Solid‐state ^11^B NMR spectroscopy further confirms the presence of S‐B bonds. Compared with the unmodified ZrB_2_ (black curve), the modified m‐ZrB_2_ (red curve) exhibits a new boron signal peak at 15 ppm, which is consistent with the characteristic position of S‐B bonds. This confirms the formation of new interfacial interactions between the PTA coating and the ZrB_2_ surface, which is consistent with the simulation results. Collectively, these results demonstrate from both theoretical and experimental perspectives that the sulfur‐containing polymer coating significantly enhances the interfacial adhesion with ZrB_2_, providing key support for achieving a robust and stable encapsulation effect (Figure 6c).

## 3 Conclusion

In summary, we have developed an advanced mechanochemical synthesis paradigm that successfully resolves the long‐standing interfacial trade‐offs of Group 13 and related fillers. By utilizing a solvent‐free mechanochemical free‐radical copolymerization route driven by mechano‐oxygen synergy, highly conformal sulfur‐containing polymer shields are constructed in situ directly onto nano‐Al, ZrB_2_, and B_4_C particles. The cornerstone of this strategy lies in its exploitation of interfacial Lewis acid‐base pairings, anchoring the organic‐inorganic boundary with ultra‐robust S–Al and S–B coordinative networks. Comprehensive spectroscopic validation and modeling collectively confirm that this localized chemical affinity dramatically reinforces interfacial adhesion while meticulously preserving the intrinsic lattice structures and functionality of the underlying fillers. The profound utility of this approach is highlighted by multi‐scenario performance breakthroughs, including an accelerated droplet burning rate (0.82 mm^2^/s) for nanofluid fuels, a 33% ablation reduction for thermal protection shields, and concurrent ∼270% elevations in both strength and toughness for structural composites. This customizable, environmentally benign platform circumvents the ecological and kinetic barriers of traditional liquid‐phase protocols, offering an efficient and sustainable pathway for next‐generation hybrid materials.

## Author Contributions

**Chen Wang**: methodology, investigation, Writing – original draft. **Xiaoyang Shao**: investigation, data curation, methodology. **Zixuan Lei**: data curation. **Ruoqing Zhao**: investigation. **Xiaozhong Bao**: validation. **Shuiping Zhou**: supervision. **Wen Ao**: funding acquisition, supervision. **Zhenhua Wang**: conceptualization, writing – review and editing, writing – original draft, funding acquisition, supervision, resources.

## Conflicts of Interest

The authors declare no conflicts of interest.

## Supporting information




**Supporting File**: advs78222‐sup‐0001‐SuppMat.docx.

## Data Availability

The data that supports the findings of this study are available in the supplementary material of this article.
