# Supplementary material for: Mechanochemical Engineered Sulfur Copolymers Onto Group 13 and Related Fillers Through Lewis Acid‐Base Pairing
Source: Adv Sci (Weinh). 2026 Oct 11:e78222. Online ahead of print. doi: 10.1002/advs.78222 (PMC13654859; doi:10.1002/advs.78222)
Supplement: Supplementary file 1 [file ADVS-9999-e78222-s001.docx]

Mechanochemical Engineered Sulfur Copolymers onto Group 13 Fillers through Lewis Acid-Base Pairing

Chen Wang#, Xiaoyang Shao#, Zixuan Lei, Ruoqing Zhao, Xiaozhong Bao, Shuiping Zhou*, Wen Ao* and Zhenhua Wang*

Table of Contents

[Experimental Procedures	3](#_Toc233215860)

[Materials	3](#_Toc233215861)

[Experimental Section/Methods	3](#_Toc233215862)

[Figure S1. Aerobic mechano-RAFT driven by ball-milling.	7](#_Toc233215885)

[Figure S2. ^1^H NMR (CDCl_3_) of reaction mixture of polymerization of AGE and TA.	8](#_Toc233215887)

[Figure S3. ^1^H NMR (CDCl_3_) of reaction mixture of polymerization of TAMe and nBA.	9](#_Toc233215889)

[Figure S4. ^1^H NMR (CDCl_3_) of reaction mixture of polymerization of TABn and nBA.	10](#_Toc233215890)

[Figure S5. ^1^H NMR (CDCl_3_) of reaction mixture of polymerization of TATF and nBA.	11](#_Toc233215892)

[Figure S6. ^1^H NMR (CDCl_3_) of reaction mixture of polymerization of NIPAM and TAMe.	12](#_Toc233215893)

[Figure S7. ^1^H NMR (CDCl_3_) of reaction mixture of polymerization of NIPAM and TABn.	13](#_Toc233215895)

[Figure S8. ^1^H NMR (CDCl_3_) of reaction mixture of polymerization of NIPAM and TATF.	14](#_Toc233215896)

[Figure S9. ^1^H NMR (CDCl_3_) of reaction mixture of polymerization of NIPAM and TADA.	15](#_Toc233215897)

[Figure S10. ^1^H NMR (CDCl_3_) of reaction mixture of polymerization of NIPAM and TABA.	16](#_Toc233215898)

[Figure S11. SEM images of nAl and m-nAl.	17](#_Toc233215900)

[Figure S12. Time-resolved fluorescence emission spectra and intensity evolution	18](#_Toc233215902)

[Figure S13. Digital photos and quantitative gray level analysis	19](#_Toc233215904)

[Figure S14. Variation of the droplet size during combustion over time.	20](#_Toc233215906)

[Figure S15. Ignition delay times of the three nanofluids	21](#_Toc233215908)

[Figure S16. SEM images of pristine B_4_C and ZrB_2_.	22](#_Toc233215910)

[Figure S17. XRD patterns of (a) nAl and m-nAl and (b) B_4_C and m-B_4_C.	23](#_Toc233215912)

[Figure S18. DSC curves of pristine and copolymer-coated (a) B_4_C and (b) ZrB_2_.	24](#_Toc233215914)

[Figure S19. FT-IR spectral analysis	25](#_Toc233215916)

[Figure S20. XPS wide-scan spectra of ZrB_2_ and m-ZrB_2._	26](#_Toc233215918)

[Figure S21. XPS wide-scan spectra of nAl and m-nAl.	27](#_Toc233215920)

[Figure S22. High-resolution Al 2p XPS spectra	28](#_Toc233215922)

[Table S1. Aerobic mechano-RAFT with different monomers	29](#_Toc233215923)

[Table S2. Quantitative calculation results of XPS.	30](#_Toc233215924)

[Table S3. Binding energy of PMA/PTA with γ-Al_2_O_3_ or ZrB_2_ interface layer.	31](#_Toc233215925)

[Author Contributions	32](#_Toc233215926)

Experimental Procedures

Materials

All chemicals were used as received unless otherwise indicated. All chemicals were used as received unless otherwise indicated. 2,2,2-Trifluoroethyl acrylate were purchased from Energy Chemical (China). Allyl glycidyl ether (AGE), E-51 epoxy resin (ER), phenolic resin (PR), zirconium boride (ZrB_2_, 10 μm), boron carbide (B_4_C, 20 μm), α-lipoic acid (TA), 4-Vinylphenylboronic acid (VPBA), 2,2,2-trifluoroethyl acrylate (FTA) and lauryl acrylate (LA) were purchased from Adamas. Hubei Institute of Aerospace Chemotechnology provided the nmAl particles and RP-3 aviation kerosene.

Experimental Section/Methods

General procedure for synthesis of Et_3_B-PyOMe

20 mmol of 4-methoxypyridine was dissolved in 20 mL anhydrous THF, and the resulting solution was purged with dry nitrogen for 15 min. After cooling the deoxygenated solution in an ice-water bath, a commercially available triethyl borane (TEB) 1 M solution in THF (20 mL, 20 mmol) was added slowly via syringe. After 1 h of stirring at ambient temperature in anitrogen atmosphere, evaporation of solvent THF under reduced pressure afforded the desired Et_3_B-PyOMe in 89% yield. ^1^H NMR (500 MHz, Chloroform-d) δ 8.39-8.20 (m, 2H), 6.96-6.76 (m, 2H), 3.86 (s, 3H), 0.51 (t, J = 7.7 Hz, 9H), 0.32 (q, J = 7.4 Hz, 6H).

General procedure for synthesis of lipoic acid derivatives

Lipoic acid trifluoroethyl ester (TATF): TA (2.27 g, 11 mmol, 1.1 equiv.) was slowly added and dissolved in 30 mL anhydrous CH_2_Cl_2_ while stirring under ice bath. 4-(N,N-dimethylamino) pyridine (DMAP, 0.12 g, 1.0 mmol, 0.1 equiv.) and N-(3-dimethylamino-propyl)- N’-ethylcarbodiimide hydrochloride (EDCI, 2.3 g, 12 mmol, 1.23 equiv.) were slowly added into the solution and followed by dropwise addition of 2,2,2-trifluoroethanol (1.0 g, 10 mmol, 1 equiv.). The reaction mixture was stirred overnight. The CH_2_Cl_2_ was removed in vacuum under reduced pressure and the yellow wax was dissolved in ethyl acetate and washed by 1 M HCl aqueous solution, de-ionized water, saturated NaHCO_3_ aqueous solution, and saturated NaCl aqueous solution three times respectively. The solvent was removed in vacuum under reduced pressure and the mixture was purified by column chromatography to obtain TATF as yellow oil (2.4 g, 8.3 mmol, 83% yeild). Methyl lipoate (TAME), lipoic acid benzoate (TABN), n-butyl lipoate (TABA), and 1,2-dithiolan-3-valeriansaeure-diaethylamid (TADA) was prepared using the same method as TATF.

General procedures for mechanochemically induced copolymerization of TA-R with Vms

Taking the RAFT copolymerization of TA and nBA under ball milling as an example. A 25 mL zirconia milling jar was used, together with two 10 mm zirconia milling balls. Prior to polymerization, the ambient temperature was maintained at 20±2°C to minimize thermal interference on the reaction. n-Butyl acrylate (3 g, 23.4 mmol), TA (2.07 g, 10 mmol), BTPA (0.079 g, 0.334 mmol) and Et₃B-MeOPy (0.346 g, 1.67 mmol) were added sequentially into the milling jar. The resulting mixture was subjected to ball milling at a frequency of 30 Hz for 1.5 h. All other copolymerization experiments were carried out following the same procedure.

Preparation of m-nAl

Modification of nAl powder was carried out via ball milling, using a 50 mL zirconia milling jar with 5 10 mm and 10 5 mm zirconia grinding balls. In the jar, 0.5 g of nano-aluminum powder, 0.5 g of TATF, 0.5 g of LA, 0.01 g BPTA were added in sequence. The milling was performed at a rotational speed of 500 r/min for 0.5 hours, with the ambient temperature of the milling process maintained at 25±2 °C. The resulting product was subsequently washed with methanol to remove unreacted monomers and then dried, affording purified modified aluminum powder with the structure m-nAl, designated m-nAl.

Preparation of m-B_4_C

Modification of boron carbide powder was carried out via planetary ball milling, using a 50 mL zirconia milling jar with ten 10 mm and twenty 5 mm zirconia grinding balls. In the jar, 3 g of boron carbide powder, 1 wt.% of lipoic acid (TA) and allyl glycidyl ether (AGE), 1 wt.% of Et_3_B-MeOPy initiator, and 0.01 wt.% of BTPA were added in sequence. The milling was performed at a rotational speed of 500 r/min for 2 hours, with the ambient temperature of the milling process maintained at 25±2 °C. The resulting product was subsequently washed with methanol to remove unreacted monomers and then dried, affording purified modified aluminum powder with the structure B_4_C@PAGE-*co*-PLA, designated m-B_4_C.

Preparation of m-ZrB_2_

Modification of zirconium boride powder was carried out via planetary ball milling, using a 30 mL zirconia milling jar with five 10 mm and ten 5 mm zirconia grinding balls. In the jar, 2 g of boron carbide powder, 1 g of lipoic acid (TA) and 1 g of 4-Vinylbenzeneboronic acid (VPBA), 1 wt.% of Et_3_B-MeOPy initiator, and 0.01 wt.% of BTPA were added in sequence. The milling was performed at a rotational speed of 500 r/min for 1 hours, with the ambient temperature of the milling process maintained at 20±2 °C. The resulting product was subsequently washed with methanol to remove unreacted monomers and then dried, affording purified modified aluminum powder with the structure ZrB_2_@PVPBA-*co*-PLA, designated m-ZrB_2_

Preparation of B_4_C/ER composite

The B_4_C/ER composite was prepared by casting method, with E-51 as the epoxy resin and isophorone diamine (IPDA) as the curing agent. The modified boron carbide, E-51 epoxy resin and curing agent were mixed and defoamed in a vacuum deaerator at 10 Pa and 2000 r/min. After mixing, the mixture was cast into a preheated mold, then pre-cured in an oven at 80°C for 2 h and subsequently fully cured at 120°C for 4 h. Finally, the cured epoxy resin was aged at 100°C for 6 days.

Preparation of ZrB_2_/PR composite

ZrB_2_ powder (5 wt.%) and phenolic resin (PR) were added together into a beaker, and thoroughly mixed by mechanical stirring for physical blending at room temperature. After mixing, the mixture was cast into a preheated mold, then placed in a blast drying oven for staged curing: successively cured at 120^o^C, 140^o^C and 160^o^C for 2 hours each, and finally cured at 180^o^C for 4 hours to obtain the target composite material.

Preparation of nanofluid fuel

First, a predetermined mass of aluminum powder was weighed and then added into kerosene, with the aluminum concentration in the fuel set at 5.0 wt.%. The mixture was vigorously stirred on a magnetic stirrer for 10 minutes to ensure thorough mixing of the two components. After stirring, the mixture was subjected to ultrasonication for 30 minutes to reduce particle agglomeration and improve the dispersion stability of the fuel. Upon completion of ultrasonication, the aluminum-based nanofluid fuel was obtained.

SEM-EDS analysis of particles

Scanning electron microscopy (SEM) was carried out using a Gemini SEM 300 (ZEISS) to examine the microstructures of the modified nAl and CCPs. Analyses of the elements on the Al@PFTA-3/7 surface were performed by Energy Dispersive Spectroscopy (EDS).

Fourier transform infrared (FT-IR) characterization

The FT-IR instrument Nicolet 560, manufactured by Thermo Fisher Scientific in the United States, was utilized to obtain the FT-IR spectra of modified Al powder. The spectral range for testing wavenumbers ranged from 4000 to 200 cm^−1^

XRD characterization

The crystal phase of Al powder was determined by X-ray Diffraction (XRD, Bruker D8 Advance, Germany), operated with Cu Kα radiation (λ = 1.5406 Å). The scan was performed in the 2θ range from 20° to 80° with a step size of 0.02° and a scan speed of 1.0°/min to obtain the diffraction pattern.

Size and zeta potential characterization

Dynamic light scattering (DLS) were commonly used to show the size contribution of modified Al powder. The DLS and Zeta potentials were calculated by Malvern Zetasizer Nano ZS.

XPS characterization

X-ray photoelectron spectroscopy (XPS) spectra were recorded by ULVAC PHI Quantera, and the profiles were analyzed using the Avantage software system.

Single droplet ignition and combustion experimental system

The droplet manipulation device is the core component of the droplet ignition and combustion experiment, where most experimental operations are carried out. It is capable of generating, fixing and igniting a single droplet. A modified micropipette was used to produce single droplets, with the droplet diameter controlled at approximately 0.5~1.5±0.1 mm in the experiment. Single droplets were suspended by silicon carbide (SiC) fiber bundles: two SiC fiber bundles were crossed at right angles, and the droplet was hung at the intersection, a method that facilitates the stable fixation of the droplet position. Droplet ignition was achieved by a heating coil placed beneath the droplet; the heating wire was made of nichrome with a resistance of approximately 1 Ω, which can provide a high-temperature environment for ignition. The droplet combustion process was recorded by a Phantom VEO 340 high-speed camera, which was set at a frame rate of 2000 fps and a resolution of 2048×1024 pixels. The high-speed camera was used to capture and record information such as droplet flame, droplet morphology and droplet diameter variation accurately and in real time. A single-frequency high-intensity lamp was used as backlight on the opposite side of the droplet to enable a more accurate determination of droplet size and clear observation of droplet micro-explosion and other phenomena. Meanwhile, an Avaspec2048-USB2 fiber optic spectrometer was employed to collect the emission spectra during droplet combustion, from which the flame spectral information was obtained; the combustion status of nano-aluminum particles was also determined via the characteristic AlO emission spectra. A switching power supply was used to send voltage signals for the synchronous triggering of all devices. A computer was utilized to set up and operate the software of each device, as well as to store the experimental data.

^1^H NMR Test

The proton nuclear magnetic resonance (^1^H NMR) spectroscopy of the samples, which were dissolved in CDCl_3_, was conducted using a spectrometer. The instrument used for this analysis was the AVANCE NEO 500 spectrometer, manufactured by Bruker in Germany.

Contact angle test

The contact angle of the modified powder was assessed at room temperature using a contact angle meter (KRUSS-DSA25, Germany).

GPC traces of copolymer

The molecular weights (*M*_n_) and *Ð* (*M*_w_/*M*_n_) were determined by Gel Permeation Chromatography (GPC). The GPC was performed in tetrahydrofuran solution at 35°C with an elution rate of 0.5 mL/min on an Agilent 1260 HPLC system equipped with a G7110B pump and a G7162A refractive index detector. The apparent molecular weights were determined on a single PL gel MIXED-C columns using linear poly (methyl methacrylate) standards.

DSC-TG characterization

Differential scanning calorimetry (DSC) and thermogravimetric analysis (TGA) tests were performed on a simultaneous thermal analyzer (TA-SDT650, US) to determine the thermal performance of samples at a heating rate of 15 K min^−1^ within the temperature range from 30°C to 1000°C. Oxygen was used as the reaction gas, at a constant flow rate of 40 mL min^-1^.

Modeling Process and Calculation of Al_2_O_3_/ZrB_2_ with PMA/PTA Simulation Experiments

To investigate the interfacial bonding interaction and interaction mechanism between the alumina layer on the surface of nano-aluminum powder (nAl)/zirconium diboride(ZrB_2_) and poly(methyl acrylate) (PMA)/polythiooctanoate (PTA) polymers, first-principles calculations were carried out in this work using *Materials Studio* software. Amorphous Al_2_O_3_ naturally formed by oxidation on nano-aluminum powder exhibits a highly similar surface structure to γ-Al_2_O_3_. Accordingly, the γ-Al_2_O_3_ model was adopted to substitute amorphous alumina on aluminum powder surfaces for modeling and computational analysis. The crystal structure of γ-Al_2_O_3_ was initially constructed using the “Build Crystal” module. The space groups of γ-Al_2_O_3_ is 167. The lattice parameters—γ-Al_2_O_3_ (a=b=4.761 Å, c=12.994 Å, α=β=90°, γ=120°)—showed good agreement with reported experimental values, confirming the validity of the constructed models. In this simulation, the (110) plane of γ-Al_2_O_3_ was selected as the crystal plane. To simulate the interface interaction more accurately, the γ-Al_2_O_3_ unit cells were cleaved along their respective crystal planes and expanded into 2 × 2 supercells to provide sufficient surface area for interaction studies. A 20 Å vacuum slab was added using the Build Vacuum Slab tool to avoid inter-layer interactions. All geometry optimizations and energy calculations were performed in the Castep module using the generalized gradient approximation (GGA) with the Perdew–Burke–Ernzerhof (PBE) exchange-correlation functional. The structures of the γ-Al_2_O_3_ models were optimized using the Broyden–Fletcher–Goldfarb–Shanno (BFGS) method for the wave functions, with an optimization convergence accuracy of 2×10^-5^/Atom for the total energy and an *E*_cut_ of 489.8 eV for the plane wave cutoff energy. For geometry optimization and energy calculations, we selected a 3×3×1 Monkhorst-Pack k-point grid.

Zirconium diboride (ZrB_2_) is a typical metallic conductive ceramic. In this work, a thermodynamically stable Zr-terminated (0001) slab model was selected as the substrate. The bulk primitive cell of ZrB_2_ was constructed via the Build Crystal module. ZrB_2_ adopts a hexagonal crystal system with the space group No. 191 (P6/mmm). The experimental standard lattice parameters were adopted: (a = b = 3.169 Å, c = 3.530 Å, α=β=90°,γ = 120°). Zr and B atoms were filled into the lattice by inputting fractional atomic coordinates, yielding a primitive cell containing 2 Zr atoms and 4 B atoms, which conforms to the stoichiometric ratio of ZrB_2_.

The thermodynamically most stable exposed (0001) crystal plane was cleaved using the Cleave Surface tool. The slab thickness was set to 7.0 Å, retaining 3 atomic layers of Zr and 2 atomic layers of B, which is sufficient to reproduce the bulk electronic properties of ZrB_2_. Two types of atomic terminations exist for the ZrB_2_ (0001) surface. By adjusting the fractional offset during cleavage, the Zr-terminated surface with outermost Zr atoms was chosen, which exhibits stronger interfacial interaction with ester-containing polymers. The cleaved primitive slab was expanded into a 2*2*1 supercell via the *Build Supercell* tool. A 20 Å vacuum slab was added perpendicular to the surface along the c-axis using the Build Vacuum Slab function to eliminate artificial electrostatic interactions between adjacent slabs. Atomic constraints were imposed on the slab: the bottom 3 atomic layers were fixed, while the top 2 surface layers were allowed to relax freely. All geometry optimizations and single-point energy calculations were carried out in the CASTEP module. The Perdew–Burke–Ernzerhof (PBE) exchange-correlation functional within the generalized gradient approximation (GGA) was employed, and the TS dispersion correction (DFT-D) was activated to accurately describe van der Waals interactions between polymer chains and the ceramic surface. Since ZrB_2_ possesses metallic electronic characteristics, the Metal option was enabled during calculations to avoid severe oscillation of self-consistent field (SCF) iterations. The Broyden–Fletcher–Goldfarb–Shanno (BFGS) algorithm was adopted for structural relaxation, with convergence criteria consistent with the above content: the total energy tolerance was set to 2.0*10^-5^ eV/atom. A uniform plane-wave cutoff energy of 390.0 eV was applied to all models. A 3*3*1 Monkhorst–Pack k-point mesh was used for slab systems, whereas only the Gamma point was sampled for isolated polymer molecules. The SCF convergence threshold was 2*10^-6^ eV/atom with a maximum of 300 SCF cycles. For metallic ZrB_2_ systems, the charge density mixing amplitude was set to 0.3 to accelerate convergence.

Polymer chains of PMA and PTA were constructed using the Build Polymer module. To simplify the calculation model, all polymer chains were built with a degree of polymerization of two (*DP* = 2). A single polymer chain was placed in a cubic vacuum cell of 20 × 20 × 20 Å to eliminate artificial intermolecular interactions induced by periodic boundary conditions. All isolated polymer structures were geometrically optimized under identical CASTEP parameters to obtain the ground-state energies of free PMA and PTA molecules.

For the construction of adsorption models, the pre-optimized PMA and PTA chains were placed above the fully relaxed Al_2_O_3_ and ZrB_2_ substrates. The initial vertical distance between the ester group of the polymer and the outermost surface atoms was set in the range of 2.5–3.0 Å, ensuring that all polymer atoms were entirely confined within the vacuum region without crossing the lattice boundary. During structural optimization of the composite systems, the original atomic constraints of the substrate were retained, while the outermost surface atoms (Al, O, Zr, B) and all polymer atoms were allowed to relax freely. The symmetry conversion prompt was rejected in all adsorption calculations to avoid artificial duplication of polymer molecules caused by broken surface symmetry.

Results and Discussion

**Figure S1.** Aerobic mechano-RAFT driven by ball-milling.GPC traces of the copolymerization of (a) nBA with different TA derivatives and (b) NIPAM withdifferent TA derivatives.

**Figure S2.** Conversion analysis by ^1^H NMR (CDCl_3_) of reaction mixture of polymerization of AGE and TA.

**Figure S3.** Conversion analysis by ^1^H NMR (CDCl_3_) of reaction mixture of polymerization of TAMe and nBA.

**Figure S4.** Conversion analysis by ^1^H NMR (CDCl_3_) of reaction mixture of polymerization of TABn and nBA.

**Figure S5.** Conversion analysis by ^1^H NMR (CDCl_3_) of reaction mixture of polymerization of TATF and nBA.

**Figure S6.** Conversion analysis by ^1^H NMR (CDCl_3_) of reaction mixture of polymerization of NIPAM and TAMe.

**Figure S7.** Conversion analysis by ^1^H NMR (CDCl_3_) of reaction mixture of polymerization of NIPAM and TABn.

**Figure S8.** Conversion analysis by ^1^H NMR (CDCl_3_) of reaction mixture of polymerization of NIPAM and TATF.

**Figure S9.** Conversion analysis by ^1^H NMR (CDCl_3_) of reaction mixture of polymerization of NIPAM and TADA.

**Figure S10.** Conversion analysis by ^1^H NMR (CDCl_3_) of reaction mixture of polymerization of NIPAM and TABA.

**Figure S11.** SEM images of nAl and m-nAl.

**Figure S12.** Time-resolved fluorescence emission spectra and intensity evolution of kerosene suspensions with pristine nAl, nAl+OA, and modified nAl (m-nAl) particles (λₑₓ = 400 nm).

**Figure S13.** Digital photos and quantitative gray level analysis of the sedimentation behavior of pristine nAl, nAl+OA, and m-nAl suspensions in kerosene over time.

**Figure S14.** Variation of the droplet size during combustion over time. (a) pure fuel, (b) fuel with pristine nAl and (c) fuel with modified nAl.

**Figure S15.** Ignition delay times of the three nanofluids

**Figure S16.** SEM images of pristine B_4_C and ZrB_2_.

**Figure S17.** XRD patterns of (a) nAl and m-nAl and (b) B_4_C and m-B_4_C.

**Figure S18.** DSC curves of pristine and copolymer-coated (a) B_4_C and (b) ZrB_2_.

**Figure S19.** FT-IR spectral analysis of pristine and copolymer-coated (a) nAl, (b) B_4_C and (c) ZrB.

**Figure S20.** XPS wide-scan spectra of ZrB_2_ and m-ZrB_2._

**Figure S21.** XPS wide-scan spectra of nAl and m-nAl.

**Figure S22.** High-resolution Al 2p XPS spectra of (a) pure nAl and (b) m-nAl; high-resolution (c) C 1s and (d) F 1s XPS spectra of m-nAl.

**Table S1.** Aerobic mechano-RAFT with different monomers

| Entry^a^ | Monomer_1_ | Monomer_2_ | Target *DP* | *Conv.* (%)^b^ | *M*_n,th_ | *M*_n,GPC_ | *M*_w_*/M*_n_ |
| --- | --- | --- | --- | --- | --- | --- | --- |
| 1 | TA | nBA | 100 | TA:38.1 nBA:42.1 | 6100 | 9100 | 1.99 |
| 2 | TA | AGE | 500 | TA:23.3 AGE:24.3 | 18900 | 15200 | 1.85 |
| 3 | TAMe | nBA | 100 | TAMe:34.9 nBA:33.3 | 5600 | 6800 | 1.85 |
| 4 | TABn | nBA | 100 | TABn:26.7 nBA:32.7 | 5300 | 6900 | 2.10 |
| 5 | TATF | nBA | 100 | TATF:50.5 nBA:39.7 | 8400 | 8000 | 1.26 |
| 6 | TAMe | nBA | 100 | TAMe:<1 nBA:2.5 | - | - | - |
| 7 | TABn | nBA | 100 | TABn:<1 nBA:7.4 | - | - | - |
| 8 | TATF | nBA | 100 | TATF:<1 nBA:6.6 | - | - | - |
| 9 | TAMe | NIPAM | 100 | TAMe:38.2, NIPAM:19.5 | 4700 | 2800 | 2.12 |
| 10 | TABn | NIPAM | 100 | TABn:33.3, NIPAM:25.3 | 5400 | 3900 | 1.91 |
| 11 | TATF | NIPAM | 100 | TATF:25.6, NIPAM:19.3 | 4200 | 4400 | 1.92 |
| 12 | TADA | NIPAM | 100 | TADA:35.5, NIPAM:45.4 | 6700 | 4100 | 1.69 |
| 13 | TABA | NIPAM | 100 | TABA:38.7, NIPAM:40.6 | 6600 | 4400 | 1.69 |

Note: ^a)^ Reaction condition:[TA-R]_0_:[Vinyl]_0_:[ATTC/BTPA]_0_:[TEB-MOPy]_0_=30:70:1:5. Ball mill (25 mL ZrO_2_ milling jar, two 10 mm ZrO_2_ grinding balls, 30 Hz). Reaction time: 1.5 h. Reaction environment without air removal.

**Table S2.** Quantitative calculation results of XPS.

|  | C  (%) | O  (%) | B  (%) | S  (%) | Zr  (%) | Al  (%) | F  (%) |
| --- | --- | --- | --- | --- | --- | --- | --- |
| nAl | 31.9 | 49.1 | - | - | - | 19.0 | - |
| m-nAl | 30.0 | 42.1 |  | 16.2 |  | 8.3 | 3.4 |
| B_4_C | 55.1 | 19.6 | 25.3 | - | - | - | - |
| m-B_4_C | 69.0 | 20.3 | 6.9 | 3.8 | - | - | - |
| ZrB_2_ | 50.0 | 38.7 | 5.6 | - | 5.7 | - | - |
| m-ZrB_2_ | 75.8 | 16.4 | 3.8 | 3.1 | 0.9 | - | - |

**Table S3.** Binding energy of PMA/PTA with γ-Al_2_O_3_ or ZrB_2_ interface layer.

| **System** | ***E*_1_/eV** | ***E*_2_/eV** | ***E*_tatol_/eV** | ***E*_bind_/eV** |
| --- | --- | --- | --- | --- |
| **Al_2_O_3_/PMA** | −6182.45 | −3243.69 | −9426.23 | 0.09 |
| **Al_2_O_3_/PTA** | -24730.52 | -5216.22 | -29947.22 | 0.48 |
| **ZrB_2_/PMA** | -68004.9317 | -3235.95117 | -71240.1426 | 0.74 |
| **ZrB_2_/PTA** | -68004.9317 | -5208.47952 | -73212.056 | 1.36 |

Author Contributions

Z. H. Wang, W. Ao and S. P. Zhou supervised the project. Z. H. Wang and C. Wang designed the experiments. C. Wang, X. Y. Shao, Z. X. Lei, R. Q. Zhao, and X. Z. Bao performed the material preparation and general characterizations.C. Wang analyzed the data and wrote the manuscript. C. Wang and R. Q. Zhao drew the schematic diagrams. All the authors discussed the results and commented on the manuscript.
